# Renal Tubular Dysgenesis: Broadening the Discussion of the Etiological Spectrum

**DOI:** 10.7759/cureus.77905

**Published:** 2025-01-24

**Authors:** Inês Paiva Ferreira, Cáudia Falcão Reis, Ana Teixeira, Paula Pires de Matos, Elisa Proença

**Affiliations:** 1 Neonatology, Centro Materno-Infantil do Norte Albino Aroso, Unidade Local de Saúde de Santo António, Porto, PRT; 2 Pediatrics, Unidade Local de Saúde do Tâmega e Sousa, Penafiel, PRT; 3 Medical Genetics, Center of Medical Genetics Doutor Jacinto Magalhães, Unidade Local de Saúde de Santo António, Porto, PRT; 4 Pediatric Nephrology Unit, Centro Materno-Infantil do Norte Albino Aroso, Unidade Local de Saúde de Santo António, Porto, PRT

**Keywords:** genetics, intensive care, kidney failure, newborn, renal tubular dysgenesis

## Abstract

Renal tubular dysgenesis (RTD) is a rare disorder characterized by impaired development of the renal tubules. It is often a fatal condition that should be considered in the differential diagnosis of neonatal kidney failure. RTD can be classified as primary (linked to deleterious variants in genes encoding renin-angiotensin system (RAS) proteins) or secondary to an underlying cause. In this case report, we present a late preterm female neonate born at 35 weeks by elective cesarean section due to oligohydramnios and fetal growth restriction. At birth, she exhibited hypotonia and features consistent with Potter sequence and developed persistent anuric kidney failure, fluid-responsive hypotension, and respiratory distress requiring non-invasive ventilation. Kidney ultrasound revealed no significant abnormalities, leading to a presumptive diagnosis of RTD, which was confirmed by histopathology. Karyotype analysis revealed 46,XX,dup(1)(q24.1q25.1), which was further confirmed by whole exome sequencing. The chromosomal abnormality did not involve RAS genes, and the remaining workup was unremarkable. Despite intensive medical management, the patient died on day 20 of life. The aim of this case report was to raise awareness of this severe kidney disorder, highlighting its atypical presentation, which lacked major cardiovascular dysfunction, showed no identifiable classic etiology despite thorough investigation, and revealed a de novo chromosomal abnormality. These findings suggest the involvement of alternative pathophysiologic mechanisms in RTD.

## Introduction

Renal tubular dysgenesis (RTD) is a rare and often fatal disorder affecting the development of renal tubules, which should be included in the differential diagnosis of neonatal kidney failure (KF) [1]. Its pathophysiology is not yet completely understood, and consequently, therapeutic options remain limited. We present an atypical case of RTD to raise awareness of this condition and to highlight the potential for alternative etiologic mechanisms in its pathogenesis.

## Case presentation

A late preterm female was born to non-consanguineous, healthy parents. There was no family history of kidney disease and no maternal history of drug exposure. The pregnancy was complicated by oligohydramnios (amniotic fluid index = 2.4 cm) noted at 31 weeks of gestation and fetal growth restriction (6.8th percentile) at 33 weeks, leading to maternal hospitalization from that point onward. Doppler parameters of the uterine and middle cerebral arteries showed no abnormalities. No urinary tract or kidney malformations were observed on fetal ultrasound. After antenatal corticosteroid therapy for lung maturation, an elective cesarean section was performed at 35 weeks. Apgar scores were 9 at the 1st and 5th minutes, and the newborn’s anthropometry was appropriate for gestational age (weight = 2190 g, 26th percentile). She presented with hypotonia and features consistent with the Potter sequence, including low-set abnormal ears, a flattened nose, arthrogryposis, and clubfeet.

Right after birth, the patient developed respiratory distress and required continuous positive airway pressure ventilation (maximum inspired oxygen fraction of 0.4, positive end-expiratory pressure 5 cmH2O), complicated by a right-sided pneumothorax treated with needle thoracentesis. She presented with fluid-responsive transient hypotension without evidence of structural cardiopathy on ultrasound examination. Additionally, anuric KF (peak serum creatinine = 5.5 mg/dL, serum urea = 123 mg/dL) was observed, accompanied by non-anion gap metabolic acidosis, hyperkalemia (maximum potassium level = 7.5 mmol/L), and hyponatremia (minimum sodium level = 124 mmol/L). Kidney ultrasound showed normal-sized kidneys with normal echogenicity.

Despite diuretic therapy, the patient experienced persistent KF with increasing signs of volume overload, requiring peritoneal dialysis (PD) from day three. The PD was compromised by catheter leaks, and the patient’s condition was further complicated by peritonitis, anasarca, and coagulopathy with lower gastrointestinal bleeding. Despite organ support and broad-spectrum antibiotics, her condition progressively worsened, and she died on day 20.

**Figure 1:**
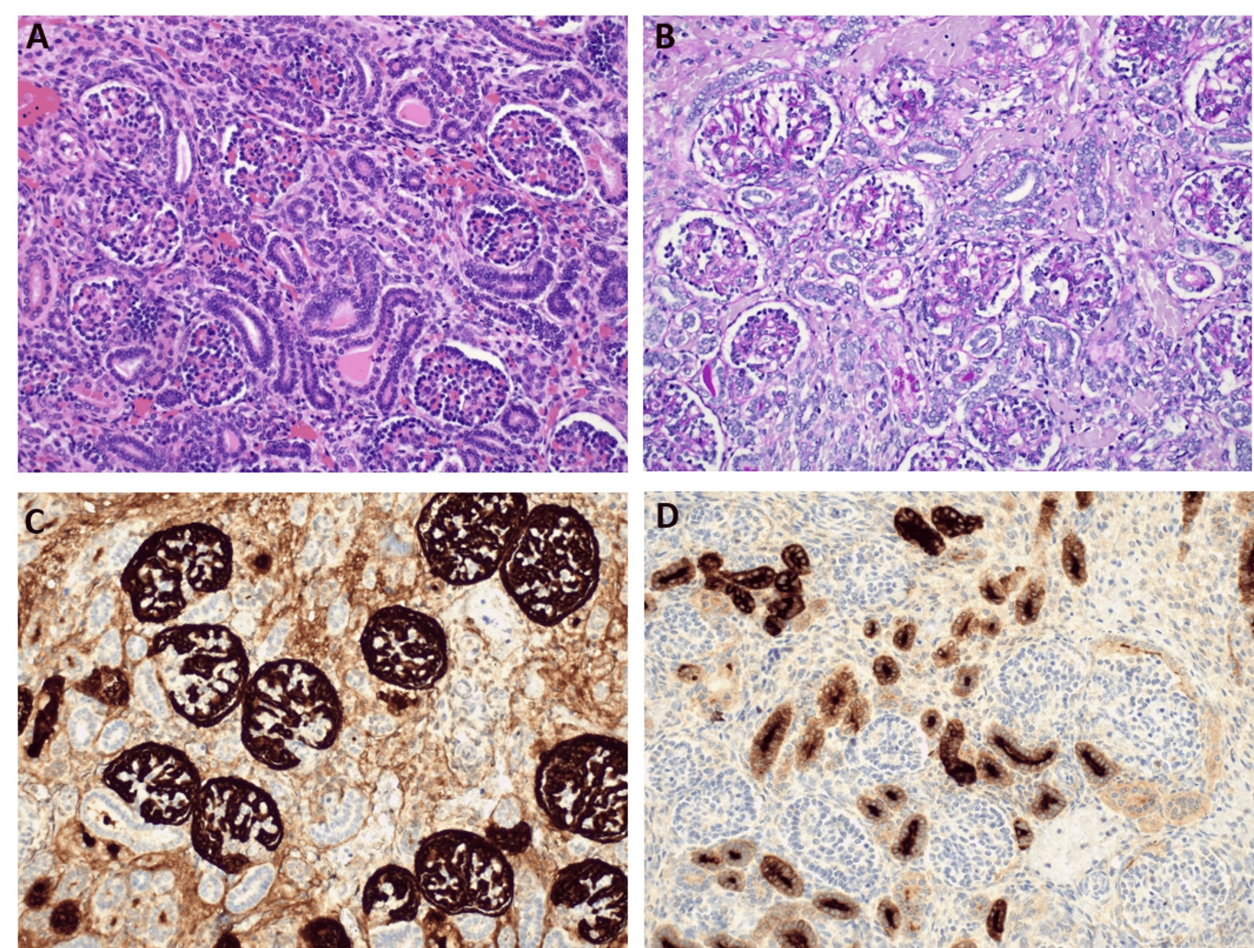
Kidney autopsy findings: pathology and immunohistochemical staining Kidney showing the presence of glomeruli and only distal tubules (A, Hematoxylin-eosin staining, 200x). Proximal tubules are not identified, as evidenced by the absence of a brush border on Periodic acid–Schiff (PAS) staining (B, PAS, 200x) and by the lack of CD10 staining in the tubules (C, CD10, 200x). Immunohistochemical analysis with epithelial membrane antigen (EMA) highlights the distal tubules (D, EMA, 200x).

Given the neonatal refractory anuric KF with structurally normal kidneys on ultrasound, a presumptive diagnosis of RTD was made. Skull ossification defects on radiography further supported this suspicion. The diagnosis was confirmed by kidney biopsy and autopsy findings showing a reduction in the number of proximal tubules (Figure 1). Classical cytogenetic analysis of peripheral blood leukocytes showed a 46,XX,dup(1)(q24.1q25.1) karyotype. The interstitial duplication of 9.3 Mb at the long arm of chromosome 1, encompassing 109 genes, was confirmed by array-comparative genomic hybridization and whole exome sequencing. This duplication had not been described before and did not contain RTD causal genes (*ACE*, *REN*, *AGT*, and *AGTR1*) or any deleterious variants. Parental karyotypes were normal, pointing toward de novo occurrence. Antemortem levels of active renin (47 pg/mL) and ferritin (80 μg/L) were within normal limits. Placental histopathology revealed a small area (1 cm) of ischemic necrosis.

## Discussion

RTD is a rare and severe disorder characterized by the absence or poor differentiation of proximal tubules [1]. It is one of several conditions that can lead to neonatal KF. Although the differential diagnosis of neonatal KF is broad, when associated with oligohydramnios, the list can be narrowed to include conditions such as RTD, bilateral renal agenesis or hypo-/dysplasia, obstructive uropathy, and autosomal recessive polycystic kidney disease [2]. The presence of additional signs, such as hypotension, respiratory insufficiency, and skull ossification defects, combined with a normal kidney ultrasound, can guide the diagnosis toward RTD. However, histological examination is required for a definitive diagnosis of this condition [1, 3].

Several etiologies of RTD have been described and can be classified as primary, linked to deleterious variants in genes encoding renin-angiotensin system (RAS) proteins, or secondary (Table 1). Although its pathophysiology is not fully understood, both groups are characterized by kidney hypoperfusion, suggesting that this may be a key pathogenic mechanism in RTD [1, 3-5].

**Table 1:** Causes of RTD RTD; renal tubular dysgenesis, RAS; renin-angiotensin system, NSAID; non-steroidal anti-inflammatory drug.

Causes of RTD
Primary: monogenic renal tubular dysgenesis
Sporadic or familial autosomal recessive mutations in genes related to RAS: angiotensinogen (*AGT*; 1q42.2), renin (*REN*; 1q32.1), angiotensin-converting enzyme (*ACE*; 17q23.3) and angiotensin II receptor type 1 (*AGTR1*; 3q24)
Secondary renal tubular dysgenesis
Maternal exposure to NSAID or ACE inhibitors during pregnancy
Twin-to-twin transfusion syndrome
Major cardiac malformation
Severe fetal kidney artery stenosis
Congenital hemochromatosis
Ischemic necrosis of the placenta

In the majority of cases, RTD is fatal either in utero or shortly after birth due to refractory hypoxia caused by lung hypoplasia, severe and refractory hypotension, and anuric KF. Beyond organ support measures, treatment evidence is limited. Vasopressin and fludrocortisone may have a potential role [6]. In primary forms of RTD, the disruption of the RAS can result in low levels of angiotensin II, a key regulator of blood pressure. Angiotensin II increases renal sodium reabsorption at the proximal tubule, stimulates aldosterone production in the adrenal cortex, and promotes vasopressin release from the posterior pituitary [7]. The administration of vasopressin and fludrocortisone can, therefore, circumvent these deficiencies. Despite the biological plausibility of these strategies, the available data remain largely empirical and show variable outcomes.

In the present case, no definitive cause was identified. The chromosomal abnormality described did not involve RAS genes, which is further supported by the absence of changes in renin activity and the lack of major hemodynamic instability. Additionally, although an area of placental ischemia was documented, its limited extent weakens the potential link between placental ischemic necrosis and RTD in our patient. The identification of a de novo sizable chromosomal abnormality, along with an atypical clinical presentation without major cardiovascular dysfunction, raises the possibility of an alternative secondary pathophysiologic mechanism for RTD in this patient. This possibility has been previously suggested in the literature [8], thus emphasizing the importance of case reporting.

## Conclusions

This case underscores the importance of considering RTD in the presence of neonatal KF with normal findings on kidney ultrasound. Furthermore, it highlights the need for a comprehensive investigation of underlying conditions to provide accurate genetic counseling for future family planning.

Lastly, raising awareness of this rare disease is crucial for addressing unmet needs, such as the lack of early prenatal diagnostic markers, which could empower families to make informed decisions. A multidisciplinary approach involving neonatologists, nephrologists, geneticists, and obstetricians is key to improving diagnosis, management, and family support.
